# Predictors of Health-Related Quality of Life and Influencing Factors for COVID-19 Patients, a Follow-Up at One Month

**DOI:** 10.3389/fpsyt.2020.00668

**Published:** 2020-07-08

**Authors:** Ke-Yang Chen, Ting Li, Fang-Hua Gong, Jin-San Zhang, Xiao-Kun Li

**Affiliations:** ^1^Department of Neurology, The Second Affiliated Hospital and Yuying Children’s Hospital of Wenzhou Medical University, Wenzhou, China; ^2^Department of Anesthesia and Critical Care, and Clinical Research Unit, The Second Affiliated Hospital and Yuying Children’s Hospital of Wenzhou Medical University, Wenzhou, China; ^3^School of Pharmaceutical Sciences, Wenzhou Medical University, Wenzhou, China; ^4^Institute of Life Sciences, Wenzhou University, Wenzhou, China

**Keywords:** COVID-19, health-related quality of life, SF-36, follow-up, influencing factors

## Abstract

**Objective:**

To survey the health-related quality of life (HRQoL) and its influencing factors among patients with COVID-19 in their first medical follow up.

**Methods:**

All patients diagnosed with COVID-19 were discharged from 12 hospitals in Wenzhou, Zhejiang from Jan 17, 2020 to Mar 20, 2020. Prospectively collected and analyzed data included demographics, clinical symptoms, comorbidity, and chest CT imaging features at the first follow up, 1 month after discharge. All patients underwent the HRQoL evaluation with the Chinese version of Short-Form 36-item questionnaire (SF-36) as well as a general condition questionnaire. Factors associated with SF-36 were constructed using linear regression. Predictors of impaired physical component summary (PCS) and a mental component summary (MCS) were identified by logistic regression.

**Results:**

SF-36 demonstrated a significant difference in HRQoL in patients with COVID-19, except in physical function (PF), when compared to the general Chinese population (*p*<0.05). The multiple linear regressions demonstrated that age was negatively associated with PF, role physical (RP), but positively associated with vitality (VT) (*p*<0.05). PF, bodily pain (BP), and role-emotional (RE) were negatively associated with the female sex (*p*<0.05). For mental health, the clinical subtypes were significant associated factors (*p *< 0.05). Length of stay (LOS) was strongly negatively associated with RE and RP, and positively associated with VT (*p*< 0.05). Logistical regression revealed that non-obese overweight (OR 3.71) and obesity (OR 3.94) were risk factors for a low PCS and female sex (OR 2.22) was a risk factor for a low MCS.

**Conclusions:**

Health-related quality of life was poor among COVID-19 patients at the 1 month follow-up. Patients suffered from significant physical and psychological impairment. Therefore, prospective monitoring of individuals exposed to SARS-CoV-2 is needed in order to fully understand the long-term impact of COVID-19, as well as to inform prompt and efficient interventions to alleviate suffering.

## Introduction

The coronavirus disease 19 (COVID-19) is an infectious disease caused by the relentless spread of the severe acute respiratory coronavirus 2 (SARS-CoV-2) from human to human, all across the world (1). In the early stages of this disease, severe acute respiratory infection symptoms frequently occur (2). Some patients rapidly develop acute respiratory distress syndrome (3), and other serious complications. In addition to the pulmonary system, COVID-19 can impact multiple other organ systems, including neurological (4), cardiovascular (5), hematopoietic (6), and psychological (7). Our understanding is evolving regarding the threats COVID-19 poses to patient quality of life, mental health and life expectancy (8).

Along with social progress and the transformation of medical care and service systems, interest in health-related quality of life (HRQoL) is increasing (9). HRQoL is defined as the subjective feeling by patients of the multifaceted effect of a disease (10). The Short-Form 36-item questionnaire (SF-36) is a popular instrument for evaluating HRQoL (11). However, no study to date has explored the psychometric performance and applicability of a Chinese version of SF-36 in assessing HRQoL in COVID-19 patients at first month follow up.

Wenzhou is located in the southeastern coast of China, which has a population of 9.3 million. Wenzhou was initially one of the worst hit cities out of Hubei Province with 504 confirmed cases due to the highest volume of mobility with Wuhan (12, 13). The objective of this study was to provide theoretical basis for the targeted development of measures to improve quality of life of patients with COVID-19, as well as to guide relevant governmental departments and to improve medical and health care service strategy in the future.

## Methods

### Study Design and Participants

This is a multicenter and cross-sectional study of patients with COVID-19 who were discharged from Jan 17, 2020 to Mar 20, 2020 at first month follow up from twelve hospital isolation wards in Wenzhou City, Zhejiang Province, China. The diagnosis of COVID-19 was based on the Chinese standard at the time (14). All patients had subsequent laboratory confirmation of SARS-CoV-2. Patients with SARS-CoV-2 infection were clinically divided into four types: mild, moderate, severe, and critical, according to a WHO–China Joint Mission report on COVID-19.

### Questionnaire Development

The questionnaire contains questions with defined response categories. A few questions asked participants to provide descriptive information. Participants were informed of the purpose, the agency conducting the research, and the privacy protection of survey. The study was approved by the Institutional Review Board of Wenzhou Medical University. Written informed consent was obtained from all participants.

### SF-36 Scores on the Evaluation of HRQoL

The Chinese version of the SF-36 was translated from the International Quality of Life Assessment (IQOLA) SF-36 Standard UK Version 1.0 (15), composed of a single item of health transition (HT) and 35 items, which can be divided into 8 subscales: (1) physical function (PF), limitations due to physical health problems (role physical, RP), (3) bodily pain (BP), (4) general health (GH), (5) vitality (VT), (6) social functioning (SF), (7) limitations due to emotional health problems (role-emotional, RE), and (8) mental health. The scores of SF-36 between 0 and 100 were assigned to each domain, with higher scores indicating more favorable functional status. The eight subdomain scores were aggregated into two summary measures: physical component summary (PCS) scores and mental component summary (MCS) scores, while a low MCS or PCS (< 50) is indicative of a poor HRQoL (16).

### Chinese Population Norm

The Chinese population norm was based on the study done by He and colleagues (17). A random sample of Chinese adults in mainland China was collected and analyzed.

### Statistical Analysis

Descriptive statistics for demographic information were calculated. The results were expressed as either the mean ± standard deviation (SD) or the categorical data were summarized as percentage of the total group. Differences in quantitative data distributions between patient subgroups were tested by Student’s t-test for normally distributed data and by Wilcoxon rank-sum test or Kruskal-Wallis test for non-normally distributed data. Linear regression analysis was performed to explore the correlation between two variables. Logistic regression analysis was used to determine factors associated with decreased PCS score and MCS score. A *p*-value threshold of <0.05 was considered statistically significant. All statistical analyses were performed using SPSS software (SPSS Inc, USA).

## Results

### Demographic and Clinical Characteristics of the Patients

Five hundred and four COVID-19 patients were enrolled in this cohort study. Among the 503 survivors, 131 did not follow up and 11 provided incomplete data. A total of 361 participants were available for analysis. Baseline characteristics of the participants were collected (**Table 1**). The study participants included 186 men (51.5%) and 175 women (48.5%), with 327 mild cases and 34 severe cases. The mean age (SD) was 47.22 years (13.03) and more than half of these patients were age 40 to 60 years. The mean body mass index (BMI) was 23.64 (3.31) and the mean LOS (SD) in hospital was 19.13 days (7.60).

**Table 1:** Socio-demographic characteristics and health situation of the study sample.

Characteristics	Subtype	Number	Percentage (%)
**Sex**	Male	186	51.5
	Female	175	48.5
**Subgroup**	Mild	327	90.6
	Severe	34	9.4
**Age, years; mean (SD)**	47.22(13.03)		
**BMI**	23.64(3.31)		
**Heart rate, (bpm)**	86.63 ± 12.8		
**Systolic blood pressure, (mmHg)**	130.91 ± 17.17		
**Diastolic blood pressure, (mmHg)**	82.97 ± 11.14		
**Length of stay (LOS)**	19.13 (7.60)		
**Age**	10–19	6	1.7
	20–29	29	8.0
	30–39	67	18.6
	40–49	106	29.3
	50–59	93	25.8
	60–69	44	12.2
	70–79	13	3.6
	80–89	3	0.8
**Smoking**	Yes	17	4.7
	No	344	95.3
**Drinking**	Yes	15	4.2
	No	346	95.8
**Chronic diseases history**	Yes	115	31.9
	No	246	68.1

### Scores of SF-36 in the Study

**Figure 1:**
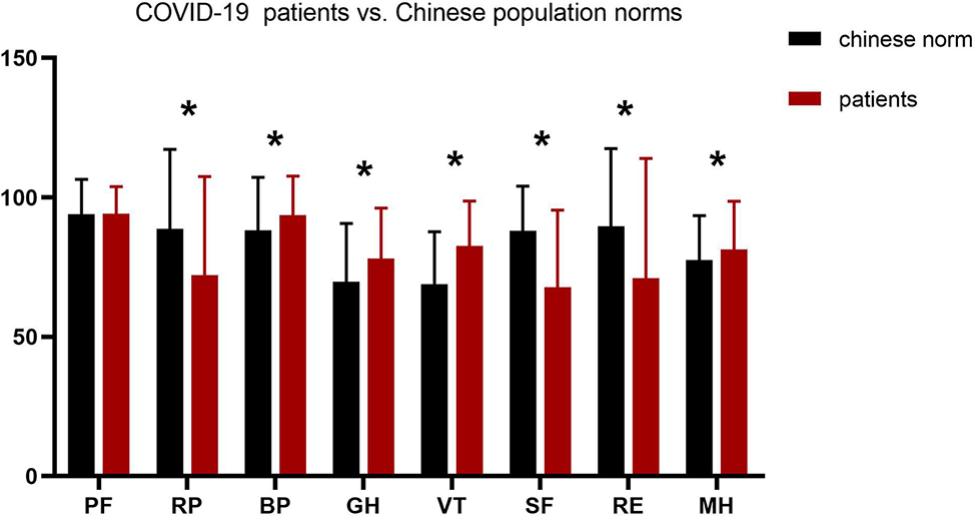
Mean scores in SF-36 for COVID-19 patients vs. Chinese population norms. PF, physical functioning; SF, social functioning; RP, role limitation due to physical problems; RE, role limitation due to emotional problems; MH, mental health; BP, bodily pain; VT, vitality; GH, general health. *p < 0.05.

The SF-36 mean score for eight specific dimensions was measured (**Figure 1**). In these eight dimensions, RP, SF, and RE subgroup scores were significantly lower in patients than the Chinese population norm (*p*<0.05). However, the scores of BP, GH, VT, and MH were higher than the norm group (*p*<0.05). Furthermore, there was no difference between two groups in PF score (*p*=0.75). At baseline, the mean scores were 55.96 ± 7.24 points for the PCSs and 48.92 ± 10.81 points for the MCSs, respectively. Comparison of HRQoL outcomes between COVID-19 patients and subjects with normal health of the different sexes was performed (**Table 2**). Compared with normative group, RP, SF, and RE subgroup scores were lower in the male group than the female group (*p*<0.01). In contrast, the scores of BP, MH, GH, and VT were higher than the normal group (*p*<0.01). However, no significant differences were observed between the two groups in PF (*p*=0.43, *p*=0.41).

**Table 2:** Comparison of health-related quality of life (HRQoL) outcomes between COVID-19 patients and subjects with normal health in different sex.

Scale	Sample	Mean± SD	*P*
PF	Male(n=186)	95.13(9.11)	0.43
	Norm	95.60(10.43)	
	Female(n=175)	93.17(10.26)	0.41
	Norm	92.57(13.88)	
RP	Male(n=186)	71.37(34.73)	<0.001
	Norm	90.76(26.09)	
	Female(n=175)	72.29(36.40)	<0.001
	Norm	86.99(30.41)	
BP	Male(n=186)	95.59(10.36)	<0.001
	Norm	89.77(17.95)	
	Female(n=175)	91.95(16.49)	<0.001
	Norm	86.73(19.84)	
GH	Male(n=186)	78.31(17.37)	<0.001
	Norm	71.20(20.03)	
	Female(n=175)	77.80(19.01)	<0.001
	Norm	68.41(21.68)	
VT	Male(n=186)	83.25(16.13)	<0.001
	Norm	70.69(17.97)	
	Female(n=175)	81.80(16.32)	<0.001
	Norm	67.30(19.36)	
SF	Male(n=186)	70.44(27.68)	<0.001
	Norm	88.39(16.20)	
	Female(n=175)	64.66(27.16)	<0.001
	Norm	87.71(15.82)	
RE	Male(n=186)	74.53(40.54)	<0.001
	Norm	91.12(26.06)	
	Female(n=175)	66.64(45.62)	<0.001
	Norm	88.15(29.52)	
MH	Male(n=186)	81.27(17.46)	<0.001
	Norm	77.80(15.78)	
	Female(n=175)	81.24(17.37)	<0.001
	Norm	77.43(17.42)	

### Factors Associated With SF-36 Among Patients in the Multivariate Linear Regression

Multivariate linear regression analysis was used to identify factors related to HRQoL of the follow-up cohort (**Table 3**). Age was negatively associated with PF, RP, but positively associated with VT (*p* < 0.05). PF, BP, and RE were negatively associated with the female sex *(p< *0.05). The severity of the clinical subtype was significantly negatively associated with the PF, GH, RE, and MH (*p* < 0.05). Length of stay (LOS) was negatively associated with RE and RP, and positively associated with VT (*p* < 0.05). In addition, there were significant negative association between lung function parameters (Forced vital capacity, FVC) and MH (*P* < 0.05).

**Table 3:** Factors associated with Short-Form 36-item questionnaire (SF-36) among patients in the multivariate analysis.

Dependent Variable	Independent Variable	*P*	Beta	95%*CI*
**PF**	Age	<0.001	−0.231	−0.250, −0.097
	Female	0.033	−0.107	−3.999, −0.174
	Clinical subtype	0.001	−0.175	−9.198, −2.442
**RP**	Chronic kidney disease	0.005	−0.147	−118.331, −21.661
	Length of stay (LOS)	0.004	−0.149	−1.167, −0.221
	Age	0.038	−0.107	−0.571, −0.016
**BP**	Female	0.013	−0.131	−6.454, −0.773
**GH**	Clinical subtype	0.042	−0.107	−13.067, −0.233
**VT**	Age	0.004	0.128	0.032,0.289
	Length of stay (LOS)	0.040	0.113	0.023,0.461
**SF**	NA	NA	NA	NA
**RE**	Length of stay (LOS)	0.002	−0.163	−1.515, −0.357
	Clinical subtype	0.014	−0.128	−33.852, −3.920
	Female	0.043	−0.105	−17.774, −0.282
	Smoking history	0.022	−0.119	−1.515, −0.357
**MH**	Clinical subtype	0.022	−0.120	−13.045, −1.012
	FVC	<0.001	−0.223	−0.052, −0.019

### Risk Factors for Low Health-Related Quality of Life

We stratified patients into two groups according to the PCS and MCS with a cutoff point of 50 and then explored the relationship between the PCS, MCS, and potential risk factors (**Tables 4** and **5**). Logistic regression analysis demonstrated that being overweight (OR 3.71, 95% CI 1.42–9.70) or obese (OR 3.94, 95% CI 1.47–10.52) were significant factors associated with a poor PCS score. Female sex (OR 2.22, 95% CI 1.30–3.81) was a significant determinant associated with an MCS < 50 in COVID-19 patients.

**Table 4:** Logistic regression analysis of COVID-19 patients with a physical component summary (PCS) < 50.

		Multivariate logistic regression results	
		OR [95% CI]	p value
**Age**	<45	1	
	45~60	2.22 [0.68, 7.17]	0.184
	>60	0.87 [0.34, 2.27]	0.780
**Sex**	Male	1	
	Female	1.84 [0.87, 1.91]	0.110
**BMI**	Normal	0.70 [0.16,2.99].	0.625
	Overweight	3.71 [1.42, 9.70]	0.008
	Obesity	3.94 [1.47,10.52]	0.006
**Clinical subtype**	Mild	1	
	Server	1.49 [0.55,4.00]	0.434
**LOS**		1.00 [0.96,1.04]	0.911
**FEV1**		0.68 [0.36,1.29]	0.235
**FVC**		1.00 [0.94,1.08]	0.925
**FEV1/FVC**		1.03 [0.99,1.06]	0.132
**Smoking**	No	1	
	Yes	0.37 [0.05,2.60]	0.319
**Drinking**	No	1	
	Yes	3.25 [0.74,14.28]	0.118
**Hypertension**	No	1	
	Yes	1.08 [0.48,2.45]	0.851
**Diabetes**	No	1	
	Yes	1.92[0.68,5.42]	0.217

**Table 5:** Logistic regression analysis of COVID-19 patients with a mental component summary (MCS) < 50.

		Multivariate logistic regression results	
		OR [95% CI]	p value
**Age**	<45	1	
	45~60	0.98 [0.44,2.20]	0.957
	>60	1.18 [0.58,2.41]	0.641
**Sex**	Male	1	
	Female	2.22 [1.30,3.81]	0.005
**BMI**	Normal	1	
	Overweight	1.14 [0.51,2.55]	0.751
	Obesity	1.26 [0.56,2.87]	0.579
**Clinical subtype**	Mild	1	
	Severe	1.70 [0.76,3.78]	0.225
**Length of stay (LOS)**		0.61 [0.27,1.36].	0.125
**FEV1**		0.79 [0.53,1.27].	0.364
**FVC**		1.00 [0.96,1.04]	0.860
**FEV1/FVC**		1.02 [0.99,1.05]	0.276
**Smoking**	No	1	
	Yes	2.16 [0.67,6.89]	0.195
**Drinking**	No	1	
	Yes	0.54 [0.16,1.85]	0.329

## Discussion

The COVID-19 pandemic is a significant psychological and physiological stressor for individuals, as well as organizations across social and economic communities worldwide. This study is the first to perform a comprehensive analysis of HRQoL in Chinese COVID-19 patients in a 1-month follow-up cohort.

In this study, we examined the absolute difference between COVID-19 patients and a normal Chinese population in SF-36 scores, including male and female subsets. Patients had higher body pain and vitality scores, but lower physiological function, social function, and role-physical scores. To our knowledge, the COVID-19 patients had uncommon symptoms, including headache, abdominal pain, and chest pain, especially in the severe/critical group (18). Therefore, the physical pain caused by the COVID-19 may last for 1 month. Furthermore, during the acute phase of the disease, patients were quarantined in hospital wards and followed strict control measures (19). They had to reduce their connection with the community. Meanwhile, they focused more on themselves and less on the individuals around them, as well as social affairs, leading to lower SF scores. These findings could be applicable to infectious disease outbreaks for informing psychosocial factors important to long-term recovery.

Multivariate analysis demonstrated that the clinical subtype was negatively correlated with PF, GH, RE, and MH. This phenomenon demonstrated that the more severe the condition of patients, the more severe the impact on physical health, as well as emotional and mental health, after hospital discharge. The results are not surprising as—in addition to the physical and psychological impairment—the long period of isolation, fear of illness, and extreme uncertainty during the COVID-19 illness had tremendous psychological and mood disturbances, such as insomnia, irritability, and anger. Recent studies observed that during the early stage of the COVID-19 outbreak, patients were at higher risk for mental health issues than the general population (20, 21). Nevertheless, the neuropsychiatric mechanism of this pandemic is currently unknown. In the brain, contiguous spread from the nasopharyngeal mucosa or a hematogenous route are two major entry pathways of SARS-CoV-2 into the CNS (22), as upper airway epithelium and vascular endothelium express Angiotensin Converting Enzyme 2 receptor (23). In addition, viruses undergo retrograde axonal transport to reach the neuron cell bodies or infecting endothelial cells of the blood-brain-barrier, epithelial cells of the blood-cerebrospinal fluid barrier in the choroid plexus (24). The breadth of this pandemic will likely require closer examination of the mechanisms underlying post-viral neuropsychiatric sequelae. Physical activity and exercise have been proven to be an effective method for directly improving both mental and physical health in general (25). Thus, COVID-19 patients with chronic diseases could also benefit from exercise.

Further subgroup analysis helped us identify patients with decreased quality of life. The PCS and MCS have been reported using norm-based scoring (mean 50 and SD 50) in nearly every published study to date (26). In our study, we found 15.5% of patients displayed poor physical health and 48.5% demonstrated poor mental health (scores <50). Multivariate logistical regression was performed to examine whether some factors were possible predictors of decreased PCS or MCS scores in SF-36. Overweight and obesity were predictors of PCSs lower than 50, indicating an association between BMI and impaired physical function. Early studies have demonstrated a similar association between increasing BMI and worse PCS scores (27, 28). BMI may influence HRQoL independent of related diseases (29). Therefore, BMI management is also crucial for the long-term rehabilitation of COVID-19. Moreover, female sex was a predictor of MCSs lower than 50, suggesting that female sex is a risk factor for the mental health quality of life in Chinese COVID-19 patients. Males and females have unique social roles and pressures, with different impacts on their disease course. Females take more care of family than males and need more energy to face stress, which results in a substantial emotional harm (30). Consequently, we argue that women are a concern in COVID-19 and should be considered for potential need for longer rehabilitation times. These findings could be applicable to infectious disease outbreaks for informing psychosocial factors important to long-term recovery. Psychotherapy such as cognitive behavior therapy and mindfulness therapy may improve the mental health of COVID-19 patients.

This study has several limitations. (1) This study may be biased due to relatively mild disease. Milder illness may correlate with higher quality of life and cause an overestimate of HRQoL. (2) Another limitation is that the study population did not include children, which should be investigated further in future research. (3) Furthermore, investigation of the physical and mental health of COVID-19 patients should include more specific, comprehensive evaluation tools, such as the Quality of Life Enjoyment and Satisfaction Questionnaire, Hamilton Anxiety Scale, and Hamilton Depression Scale, which may add to the accuracy of assessment of mental health status. (4) Finally, the cross-sectional nature of the data precludes making causal inferences.

## Conclusion

Our study provides a database for the physical profile, psychological profile, and HRQoL status of patients with COVID-19 at first month follow up. The HRQoL impairment of Chinese COVID-19 patients was significant. We propose early measures should be taken to prevent mental health problems, as well as initiation of a comprehensive program to assist COVID-19 patients in recovering basic function. Furthermore, we encourage the biomedical research community to pursue longitudinal monitoring of neuropsychiatric symptoms and status. Further follow-up is needed to assess the HRQoL of COVID-19 patients.

## Data Availability Statement

The raw data supporting the conclusions of this article will be made available by the authors, without undue reservation.

## Ethics Statement

The studies involving human participants were reviewed and approved by Wenzhou Medical University. Written informed consent to participate in this study was provided by the participants’ legal guardian/next of kin.

## Author Contributions

K-YC, J-SZ, and X-KL conceived the study. K-YC, TL, and F-HG collected and analyzed the data. K-YC, J-SZ, and X-KL wrote the paper. All authors contributed to the article and approved the submitted version.

## Funding

The study was partially supported by grants from China Academy of Engineering 2020-XY-88(2020-KYGG-04-02) and COVID-19 Emergency Response projects (No. ZY202002 and No. 2020-004).

## Conflict of Interest

The authors declare that the research was conducted in the absence of any commercial or financial relationships that could be construed as a potential conflict of interest.

## Abbreviations

BP, bodily pain; GH, general health; HT, health transition; MCS, mental component summary; MH, mental health; PCS, physical component summary; PF, physical function; RE, role-emotional; RP, role physical; SF, social functioning; VT, vitality.
